# MISEV2023: Shaping the Future of EV Research by Enhancing Rigour, Reproducibility and Transparency

**DOI:** 10.1038/s41417-024-00759-7

**Published:** 2024-03-14

**Authors:** Mark Samuels, Georgios Giamas

**Affiliations:** Department of Biochemistry and Biomedicine, School of Life Sciences, University of Sussex, JMS Building, Falmer, Brighton, BN1 9QG UK

**Keywords:** Cancer models, Biological models

Extracellular vesicles (EVs) are an area of growing interest due to their strong potential as biomarkers and therapeutics. Defined as lipid bilayer membrane-delimited particles, which are non-replicative and nano- to micro-sized, EVs are released by all cell types in every organism and have diverse roles in both normal physiology and disease. Despite the challenges associated with the study of EVs, the number of EV-related publications has been growing every year, necessitating the standardisation and proper reporting of the methodologies used for their study. In line with this, the International Society of Extracellular Vesicles (ISEV) published the Minimal Information for Studies of Extracellular Vesicles in 2014 (MISEV2014) [1], and later, an updated version in 2018 (MISEV2018) in an attempt to improve rigour and standardisation in EV studies across different groups [2].

The most recent iteration, MISEV2023, has now been published and gives a position statement reflecting the consensus of the field after surveying approximately 1000 members of ISEV [3]. The inclusion of the percentage of survey respondents that agreed or disagreed with each section showed a remarkable agreement on most topics (Table 1). An average of around 97% of respondents completely or mostly agreed with each section, with less than 1% of respondents disagreeing, and the remainder stating that they have no opinion or expertise. Although the consensus across ISEV members is clear, historically, and today, published EV studies have considerable dissimilarity in rigour, reporting of methodological details and nomenclature.

**Table 1:** The MISEV2023 survey results.

Section	Completely agree	Agree mostly	Disagree mostly	Completely disagree	No opinion and/or expertise
**1. An Introduction to ISEV and MISEV**	89.3%	10.7%	0.0%	0.0%	0.0%
**2. Nomenclature**	79.5%	19.9%	0.4%	0.0%	0.2%
**3. Collection and Pre-Processing**	70.4%	28.5%	0.1%	0.0%	1.0%
**4. EV Separation and Concentration**	74.4%	24.8%	0.1%	0.0%	0.6%
**5. EV Characterisation**	72.3%	27.0%	0.3%	0.0%	0.4%
**6. Technique-Specific Reporting Considerations**	70.6%	27.5%	0.4%	0.0%	1.5%
**7. EV Release and Uptake**	69.6%	24.3%	0.2%	0.0%	5.8%
**8. Functional Studies**	71.1%	25.1%	0.3%	0.1%	3.4%
**9. EV Analysis in Vivo**	65.5%	21.6%	0.1%	0.0%	12.7%
**Average**	73.6%	23.3%	0.2%	0.0%	2.8%

998 unique ISEV member responses were collected by the survey. For each section, ISEV members were asked their opinion and to what extent they agree or disagree. This table shows the percentage of respondents who gave each answer and the mean level of agreement across the different sections.

EVs have gained a lot of attention in recent years due to their strong biomarker and therapeutic potential as well as their ability to mediate intercellular signalling. EVs were initially reported as procoagulant particles in plasma in 1946 [4] and later as ‘platelet dust’ in 1967 [5]. Later research analysed EVs from conditioned cell culture media [6] and bovine serum [7] where ultrastructural studies revealed that these vesicles were formed through the fusion of multi-vesicular bodies with the plasma membrane [8]. Following this, these particles were shown to be antigen-presenting [9]. In 2006, embryonic stem cell-derived vesicles were suggested to horizontally transfer mRNA and proteins [10], while in 2007, it was reported that they could transfer non-coding RNAs [11]. Since then, EV-related publications have been expanding exponentially, with tens of thousands of EV studies conducted to date.

Given the challenges associated with EV study, particularly in the early 2000s, there was little rigour and standardisation in exploring this new field. The formation of the International Society for Extracellular Vesicles (ISEV) in 2011 was a first step in creating uniformity, sharing research ideas and providing guidance for EV research. In 2014, MISEV was published to ‘sensitise’ researchers, editors and reviewers to the specific reporting and experimental requirements of EV research [1]. Due to the nature of EV investigation, proper controls and reporting requirements were necessary to ensure conclusions were fully supported by data and to enable reproducibility across different research groups. For instance, different quantification techniques inevitably gave different results, therefore these needed to be reported in detail. Additionally, biological activity may be attributed to EVs, however co-segregating material may be responsible for this activity instead of EVs themselves, therefore the EV separation methods used must be described in detail.

The inability to fully separate EVs from biofluids and cell conditioned media is an important issue. As EV samples contain co-isolated materials (including RNAs, proteins and lipids), accrediting functional effects to EVs alone is challenging without proper controls. Rigorous control experiments must therefore be conducted to ascribe activity specifically to EVs rather than other secreted factors. Given the high level of interest in EVs and the ease of overinterpreting results from EV experiments, particularly for those new to the field, the MISEV publications are an invaluable resource and provide an excellent introduction to EV research for anyone interested in the topic.

MISEV2023 builds on previous guidelines and contains sections giving recommendations for EV study, reporting and nomenclature which is applicable to EV researchers, reviewers and editors, as well as anyone with an interest in the study of EVs [3]. As with previous iterations, MISEV2023 details the nomenclature of EVs and EV subtypes. It encourages the use of the term extracellular vesicle for lipid bilayer-delimited particles released from cells rather than terms such as ‘exosome’ or ‘microvesicle’ where biogenesis cannot be demonstrated, as is the case in most functional studies, in order to avoid confusion and overinterpretation of results. In a section regarding collection and pre-processing, it gives specific recommendations related to different EV sources, in parallel with some general guidance. It particularly emphasises the importance of reporting the source and storage conditions of materials as well as implementing the correct quality control measures, such as depleting material of cells prior to storage. When discussing EV separation and concentration, MISEV2023 describes the reporting requirements for the different methods of EV separation. It notes that co-isolated materials may be either contaminants or the EV corona, where molecules adsorb to the EV surface [12, 13]. The corona itself may have functional or biomarker significance, therefore reporting details of the methods in sufficient detail to allow replication of the separation is essential as the method used will affect the degree, to which, the corona remains associated with the EVs. For EV characterisation, MISEV2023 recommends approximating EV number, the degree of contamination with co-isolating components and providing an estimate of the limit of detection of the technique used to measure EVs. Orthogonal measurements are recommended as well as making available the whole distribution of particle sizes.

MISEV2023 also details several technique-specific considerations, pertaining to different microscopy techniques, protein and nucleic acid analysis and nanoparticle tracking analysis [3]. For studies analysing EV release and uptake, MISEV2023 emphasises the importance of reporting ratios of EVs and recipient cells and incubation conditions. It also notes that it is important to understand off-target effects of genetic and pharmacological manipulations to EV secretion on other processes and that these manipulations may alter one EV biosynthesis pathway; however, this may lead to changes in other EV release pathways. For functional studies, the recommendations are to perform dose-response and time course assays, to use appropriate negative EV controls and evaluate non-EV negative controls before suggesting an EV-specific role for an activity. For in vivo work, all details should be reported to allow replication. Researchers should be aware that EV biogenesis inhibitors will have off-target effects and that labelling may alter EV distribution, function and pharmacokinetics.

MISEV2023 takes a form similar to previous iterations of the MISEV guidelines. The paper summarises itself as ‘a handful of questions’ encouraging researchers to report the terms they are using and their definitions, the source of EVs, the processing of the EVs, the confidence the researcher has that the function or biomarker is related to EVs and not other components and finally if the researcher has shared enough data and details about the methods to enable reproduction of the results [3]. MISEV2023, along with previous iterations will provide guidance to researchers, reviewers, and editors in an attempt to ‘increase rigor, reproducibility and transparency’ in the study of EVs [3]. As the field is advancing quickly, it is necessary to update these guidelines regularly to provide researchers with state-of-the-art recommendations of their peers. Although ISEV membership is growing and there is broad consensus across those surveyed, the majority of EV papers do not cite MISEV publications or adhere to the reporting requirements [14]. It is therefore essential that the consensus of the field is disseminated to EV researchers.

The MISEV2023 guidelines provide a robust framework for standardising the study of EVs and their reporting in publications. Overwhelming agreement across EV researchers highlights the desire for increased transparency and rigour in the field of EV research. Researchers are prompted to question their own methods and to understand the importance of clarity and reproducibility. Therefore, it is important that EV researchers endorse and embrace MISEV2023 in a commitment to scientific integrity so that we can move forward cohesively.
